# Protective Effects of a Red Orange and Lemon Extract (RLE) on the Hepatotoxicity Induced by Ochratoxin A in Rats

**DOI:** 10.3390/antiox13030289

**Published:** 2024-02-27

**Authors:** Consiglia Longobardi, Sara Damiano, Emanuela Vaccaro, Gabriele Ballistreri, Brunella Restucci, Orlando Paciello, Salvatore Florio, Roberto Ciarcia

**Affiliations:** 1 Veterinary Medicine and Animal Productions Department, University of Naples “Federico II”, 80137 Napoli, Italy; consiglia.longobardi@unina.it (C.L.); emanuela.vaccaro@unina.it (E.V.); restucci@unina.it (B.R.); paciello@unina.it (O.P.); florio@unina.it (S.F.); rciarcia@unina.it (R.C.); 2 Council for Agricultural Research and Economics (CREA), Research Centre for Olive, Fruit and Citrus Crops, Corso Savoia, 190, 95024 Acireale, Italy; gabriele.ballistreri@crea.gov.it

**Keywords:** anthocyanins, ochratoxin A, oxidative stress, liver, red orange and lemon extract, hepatotoxicity

## Abstract

Ochratoxin A (OTA) is a highly potent mycotoxin that contaminates many kinds of food and feed sources. Its significant impact on human health and animal productivity makes it a topic of particular concern. The role of specific bioactive compounds used as dietary antioxidants is believed to be substantial due to their capacity to act as free radical scavengers. Because of the well-known oxidative stress induced by OTA, the primary objective of this work was to evaluate the antioxidant effects of a standardized powder extract recovered from citrus processing waste, red orange and lemon extract (RLE), on liver damage induced by OTA in a rat model. This study aimed to examine the impact of oral administration of RLE (90 mg/kg b.w.) on hepatic function and oxidative balance in Sprague–Dawley rats (*n* = 6/group) treated with OTA (0.5 mg/kg b.w.) over a period of 14 days. The administration of OTA alone resulted in both biochemical changes and an imbalance in redox status in the liver. However, the use of RLE alleviated the activity of antioxidant enzymes and dramatically decreased the serum levels of ALT (alanine aminotransferase), AST (aspartate aminotransferase), and ALP (alkaline phosphatase), providing evidence of its protective benefits. Based on the findings from liver histology tests, the administration of RLE resulted in mitigation of lymphoplasmacytic inflammation, steatosis, and necrosis in the OTA group. These results indicate that the novel phytoextract RLE holds potential for application in the field of nutraceuticals.

## 1. Introduction

Ochratoxin A (OTA) is a mycotoxin of significant toxicity, frequently detected in agricultural commodities and, hence, presenting a substantial global threat to the health of animals and humans [1]. The primary species responsible for producing OTA are *Aspergillus* and *Penicillium*, known for their extensive geographic assortment, with a particular prevalence in places characterized by high humidity and temperature [2]. Multiple studies have demonstrated that the main pathway of exposure to OTA in both humans and animals is through dietary consumption, as it is present in a wide variety of meals and animal feeds [3]. Regrettably, the ingestion of this mycotoxin leads to a range of adverse effects that are dependent on the dosage, with the most significant outcomes being nephrotoxicity and carcinogenicity [4,5]. Furthermore, it has been observed that OTA induces oxidative stress (OS) and membrane peroxidation at the cellular level [6]. Additionally, it impairs mitochondrial respiration, leading to the disruption of calcium homeostasis and interference with oxidative phosphorylation [7,8]. Consequently, because of its extensive dissemination and significant detrimental consequences, numerous health organisations across various nations have established guidelines specifying the maximum permissible levels of OTA in food and feed items. Starting from 1 January 2023, the European Union (EU) has implemented additional reductions to the maximum allowable levels of OTA in sultanas, soluble and roasted coffee, as well as bakery items. Furthermore, this regulation also established the maximum allowable concentrations of OTA for novel substances that were not previously addressed in the existing legislation. These substances include dried fruit (except dried grapes), some liquorice-based products, dried herbs, specific materials used in herbal infusions, and pistachios, among others (Commission Regulation (EU) 2022/1370 (2022)). The information regarding the permissible amounts of OTA is based on recent assessments conducted by the EFSA (European Food Safety Authority), focused on the toxicological risks associated with OTA and the current levels of exposure experienced by animals and consumers [3].

In recent years, there has been an increasing emphasis on the identification of various approaches aimed at reducing the contamination of food with OTA mycotoxin [9]. Currently, there is a lack of a universally accepted technique for the thorough detoxification of OTA. One of the established approaches for addressing the detrimental impacts of food and feed contaminated with OTA involves the utilisation of various bioactive compounds known to possess significant dietary antioxidant properties, as they are capable of effectively neutralising free radicals and maintaining the balance in oxidoreductive processes [10].

Among them, anthocyanins (ANTs) have been extensively studied for their properties of mitigating lipid peroxidation and preserving cell integrity. Their antioxidant potency is determined by their distinct chemical structure, which can be modified by manipulating chemical groups on the aromatic ring [11]. Among the various types of ANTs, cyandine-3-glucoside has been identified as one of the most potent, and its effectiveness can be largely attributed to its ability to form complexes with divalent metal ions that are essential for ROS production via the Fenton reaction [12].

Citrus fruits are abundant in a variety of bioactive substances that provide significant nutritional value, including ANT. Italian citrus processing facilities annually handle almost one million tonnes of citrus fruits. This activity generates a substantial quantity of waste, and failure to recycle this waste would result in significant expense. Red (blood) oranges have high concentrations of anthocyanins, with cyanidin-3-glucoside and cyanidin-3-6″-malonyl-glucoside accounting for around 95% of these compounds. Lemon fruit is also rich in flavanones and other polyphenols [13]. All these chemicals offer health benefits in the form of dietary antioxidants, taking into account the values of recycling and the promotion of a circular economy.

The liver is an essential organ that performs a variety of functions, such as the detoxification of xenobiotics and constant oxidation of fatty acids. Although the kidney is recognized as the principal organ affected by OTA, it is worth noting that OTA also induces liver impairment, as discussed in the literature by EFSA (2020) and other studies [14]. Limited research has been conducted on the hepatotoxic effects of OTA and the hepatoprotective qualities of antioxidant compounds. This indicates the need to address the current knowledge gap, particularly considering the liver’s crucial role in the biotransformation and detoxification of mycotoxins.

Hence, the objective of this work was to evaluate the scavenging power of a naturally derived extract, specifically red orange and lemon extract (RLE), rich in polyphenols derived from red orange processing wastes and other flavanones obtained from lemon peel [13], in mitigating hepatotoxicity induced by OTA in a rat animal model.

## 2. Materials and Methods

### 2.1. Ethics Statement

The utilization and treatment of animals in the current investigation received approval from the Institutional Animal Care and Ethics Committee (Approval Number: 487/2018-PR) and followed the regulations outlined in EU Directive 2010/63/EU.

### 2.2. Chemicals and Reagents

OTA was supplied by Sigma-Aldrich (Milan, Italy). Red orange and lemon extract (RLE) was obtained from the Council for Agricultural Research and Economics (CREA), Research Centre for Olive, Fruit, and Citrus Crops, (Acireale, Italy) and was produced using a patented extraction process (Italian Patent No. 102017000057761). The specific identification and relative concentrations of individual flavanones and anthocyanins derived from red orange and citrus processing wastes have been previously described [15]. In particular, the relative composition (%) by class of compounds for RLE is as follows: total flavanones (as hesperidin equivalents), 15.91 ± 0.01; total anthocyanins (as cyanidin 3-glucoside equivalents), 2.66 ± 0.01. Superoxide dismutase (SOD, Item No. 19160), malondialdehyde (MDA, Item No. MAK085), glutathione peroxidase (GPx, Item No. 38185), and catalase (CAT, Item No. CAT100) assay kits were purchased from Sigma-Aldrich (Milan, Italy). The Charles River Laboratories (Milan, Italy) supplied the animals.

### 2.3. Experimental Design and Sample Collection

A total of twenty-four male rats of the Sprague–Dawley strain, 10 weeks old (250–270 g), were randomly divided into four experimental groups (6 rats each) and housed in cages under standard conditions (20 ± 2 °C; 12 h day/night cycles), receiving a standard diet ad libitum. Animals were treated daily for 14 days by gavage as follows: control group, 1 mL sodium bicarbonate solution; OTA group, 0.5 mg/kg b.w.; RLE group, 90 mg/kg b.w.; OTA + RLE group, 5 mg/kg b.w. OTA and 90 mg/kg b.w. RLE. OTA and RLE were dissolved in 1 mL solution of sodium bicarbonate. The dose of RLE and OTA to be administered, together with the duration of the experiment, was based on prior experiment [16]. At the end of the experiment, rats were anesthetized with 2% isoflurane (Isotec 4, Palermo, Italy). After complete sedation, blood samples were collected from the abdominal aorta into non-heparinized vials and aliquoted for biochemical analysis. Each rat was then euthanized, and the liver was removed and divided into aliquots to quantify the OS markers and lipid peroxidation, as well as to be adequately prepared for histology analysis.

### 2.4. Serum Hepatic Biomarkers Determination

Alanine aminotransferase (ALT), aspartate aminotransferase (AST), and alkaline phosphatase (ALP) were measured in sera after the treatment using an automatic analyzer (PKL PPC 125, Paramedical srl, Salerno, Italy). The results were reported in units per liter (U/L). Total protein concentrations were colorimetrically determined in the collected samples via Bradford assay (Biorad Protein Assay, Biorad, Milan, Italy).

### 2.5. Determination of Malondialdehyde Levels and SOD, CAT and GPx Activities

MDA levels were measured according to Ohkawa et al. [17]. Briefly, tissue homogenate was mixed with an aqueous solution of 30% thiobarbituric acid. After heating at 95 °C for 60 min, the red pigment produced was extracted with *n*-butanol–pyridine mixture and its absorbance was read at 532 nm with a spectrophotometer (Glomax Multi detection system, Promega, Milano, Italy). Data were expressed as nanomoles of MDA per milligram of proteins.

To assess SOD, CAT, and GPx activities, tissue homogenates were centrifuged at 10,000× *g* for 15 min at 4 °C, and the supernatants were tested according to a previous study [16]. Enzymatic activities were expressed as units per milligram of protein (U/mg of protein).

### 2.6. Histological Investigations

During the necropsy procedure, the liver sections were appropriately collected for histology. In particular, organs were fixed in Bouin solution for 24 h and, after dehydration in ascending ethyl alcohol, embedded in paraffin. Slices measuring 3 μm were stained with hematoxylin and eosin (HE) and Masson’s trichrome (MTRC). At the end of the process, images were acquired with a Pannoramic scan II (3 Dhistech, Budapest, Hungary, 2023). The hepatic lesions were assessed by examining at least 10 microscopic fields at 40× magnification for each section obtained from each animal. The average for each animal was scored using an already defined scoring system [18,19]. Inflammation was scored as follows: score 0, no inflammatory foci; score 1 (mild), <2 foci per 40× field; score 2 (moderate), 2–4 foci per 40× field; score 3 (severe), >4 foci per 40× field. The extent of the steatosis was scored as follows: score 0, <5% hepatocytes; score 1 (mild), 5–33%; score 2 (moderate), >33–66%, score 3 (severe), >66%. The extent of the necrosis was scored as follows: score 0, 0% necrotic hepatic tissue; score 1 (mild), <10%; score 2 (moderate), >10–50%, score 3 (severe), >50%. Fibrosis was graded 0 to 3 (0, absent; 1, perisinusoidal/pericellular fibrosis; 2, periportal fibrosis; 3, bridging fibrosis). Furthermore, the presence or absence of sinusoidal dilation and central vein dilation was recorded for each case.

### 2.7. Statistical Analysis

Statistical analyses were performed using GraphPad (GraphPad Software version 8.00, GraphPad Software Inc., San Diego, CA, USA). MDA, SOD, CAT, and GPx assays, conducted on 6 animals/group, were analyzed via analysis of variance (ANOVA) followed by Tukey’s test. Histologic semiquantitative scores as well as the frequency of fibrosis, sinusoidal dilation, and central vein dilation were evaluated using the Mann–Whitney test. Statistical significance was attributed to values of *p* < 0.05.

## 3. Results

### 3.1. RLE Effects on Hepatic Enzymes and Serum Total Protein Levels in Rats

The liver functionality assessment involved the measurement of ALT, AST, ALP, and the total protein levels in the sera of rats (Table 1). The OTA group exhibited a substantial rise in ALT (+143.10%), AST (+59.25%), and ALP (+273.33%) activity levels in respect to the control group (*p* < 0.0001), accompanied by a decrease in the total protein concentration (−39.44%, *p* < 0.0001). In contrast, the co-treatment with RLE (OTA + RLE) resulted in statistically significant reductions in ALT, AST, and ALP activities by 22.81%, 16.78%, and 32.89% (*p* < 0.0001), respectively. Additionally, this co-treatment led to a considerable decrease in the serum total protein concentration by 70.49% (*p* < 0.0001).

### 3.2. RLE Co-Treatment Alleviated Lipid Peroxidation in the Liver of OTA-Intoxicated Rats

**Figure 1:**
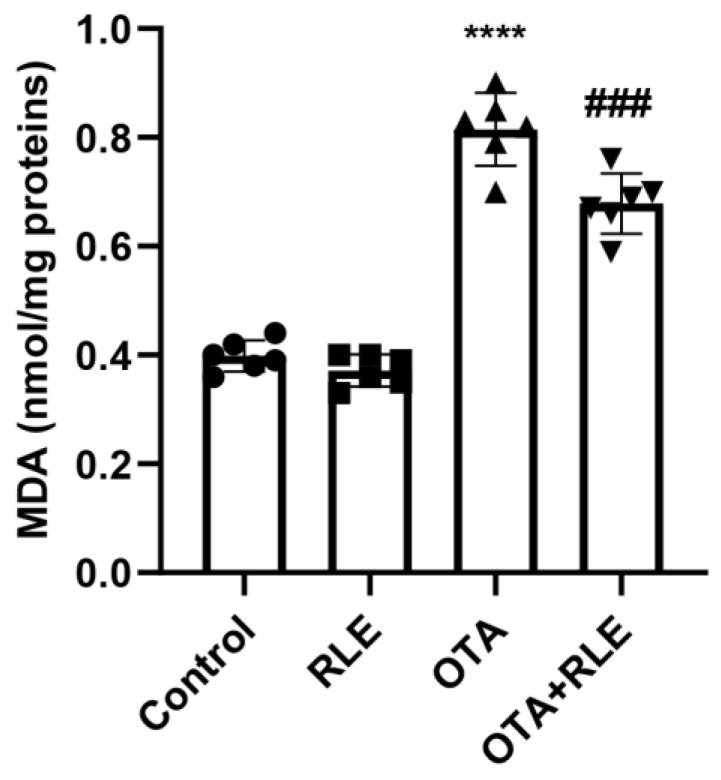
Lipid peroxidation expressed in nanomoles of MDA per milligram of proteins (nmol/mg proteins) in rat liver. Control group (control); RLE group (RLE); ochratoxin A group (OTA); OTA plus RLE group (OTA + RLE). Results are expressed as mean ± standard deviation (SD) of *n* = 6 rats (**** *p* < 0.0001 vs. control; ^###^ *p* < 0.001 vs. OTA).

MDA, a widely used marker for lipid peroxidation, was investigated in all the experimental groups. The results revealed a significantly increase in the OTA group (0.81 ± 0.067) when compared with the control (0.39 ± 0.028) (*p* < 0.001). The group receiving OTA + RLE showed a marked decrease in MDA concentration in comparison to the group receiving only OTA (*p* < 0.0001). Indeed, the MDA value shifted from 0.81 ± 0.067 (OTA) to 0.67 ± 0.056 (OTA + RLE) (*p* < 0.001), while MDA levels during RLE administration alone (0.37 ± 0.029) were comparable to the control rats (0.39 ± 0.028) (Figure 1).

### 3.3. RLE Mitigates SOD, CAT, and GPx Activities during OTA Intoxication

**Figure 2:**
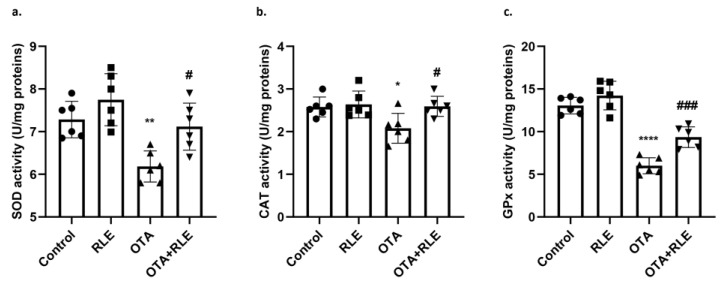
(**a**) Liver SOD activity; (**b**) liver CAT activity; (**c**) liver GPx activity. Control group (control); RLE group (RLE); ochratoxin A group (OTA); RLE plus OTA (RLE + OTA). Data are expressed as mean ± standard deviation (SD) of *n* = 6 rats (* *p* < 0.05, ** *p* < 0.01, **** *p*<0.0001 vs. control; ^#^ *p* < 0.05, ^###^ *p* < 0.001 vs. OTA).

Figure 2a–c shows the levels of SOD, CAT, and GPx in the livers of the animals belonging to the four experimental groups. SOD, CAT, and GPx activities were found to be considerably lower in the livers of OTA rats in comparison with the control group. In particular, SOD values exhibited a drop from 7.28 ± 0.43 (control) to 6.18 ± 0.36 (OTA) (*p* < 0.01), CAT shifted from 2.57 ± 0.23 (control) to 2.07 ± 0.35 (OTA) (* *p* < 0.05), while GPx declined from 13.0 ± 0.97 (control) to 6.0 ± 0.94 (OTA) (*p* < 0.0001). Co-treatment with RLE statistically increased SOD, CAT, and GPx activities with respect to the OTA intoxicated group. In fact, the SOD value was 7.11 ± 0.55 in the OTA + RLE group, compared with 6.18 ± 0.36 in the OTA group (*p* < 0.05); the CAT value was 2.59 ± 0.23 in the OTA + RLE group, compared with 2.07 ± 0.35 in the OTA group (*p* < 0.05); the GPx value was 9.35 ± 1.20 in the OTA + RLE group, compared with 6.0 ± 0.94 in the OTA group (*p* < 0.001). Administration of RLE alone did not show any significant impact on SOD, CAT, or GPx activities with respect to the control group. In fact, the SOD value was 7.74 ± 0.61 in the RLE group, compared with 7.28 ± 0.43 in the control group; the CAT value was 2.64 ± 0.31 in the RLE group, compared with 2.57 ± 0.23 in the control group, and the GPx value was 14.2 ± 1.68 in the RLE group, compared with 13.0 ± 0.97 in the control group.

### 3.4. Mitigation of Toxic Effects of OTA in Livers’ Histological Sections

**Figure 3:**
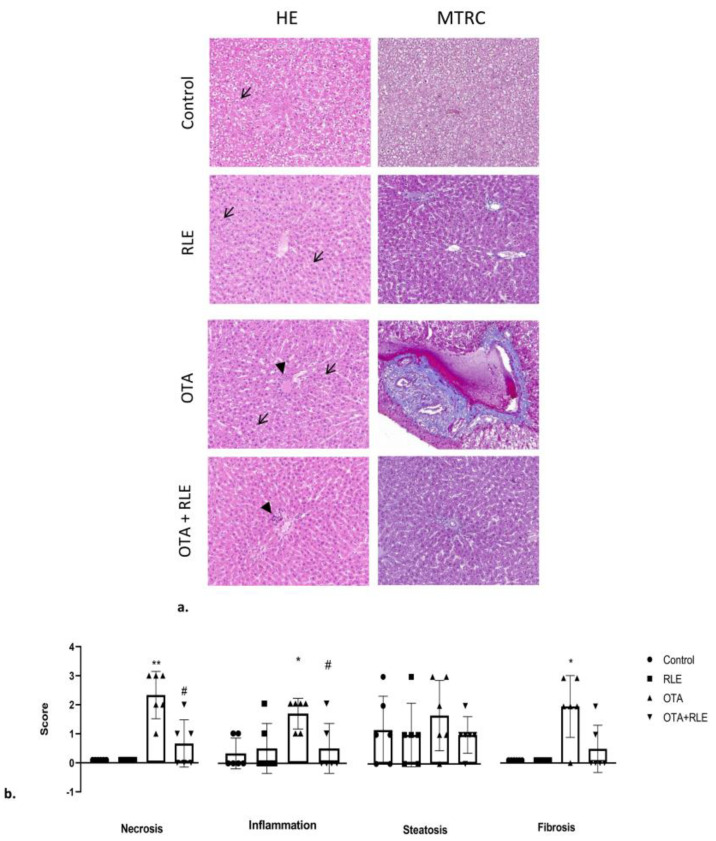
Rats’ liver with hematoxylin and eosin (HE) and Masson’s trichrome (MTRC) stains, 40× magnification, scale bar = 0.020 mm. Control group (control); red orange and lemon extract group (RLE); ochratoxin A group (OTA); ochratoxin A plus red orange and lemon extract group (OTA + RLE). (**a**) Rats in the control and RLE groups showed numerous disseminated swollen hepatocytes with intracytoplasmic optically empty vacuoles (steatosis, arrow) with HE staining, and a normal amount of interstitial connective tissue (blue) with MTRC staining. OTA-treated rats showed periportal infiltration of lymphocytes (arrowhead) and numerous disseminated swollen hepatocytes with intracytoplasmic optically empty vacuoles (steatosis, arrow). Rats in the OTA group also showed portal spaces severely expanded by abundant fibrous connective tissue (blue) with MTRC staining. Rats in the OTA + RLE group showed focal periportal infiltration of lymphocytes (arrowheads) and numerous disseminated swollen hepatocytes with intracytoplasmic optically empty vacuoles (steatosis, arrow) with HE staining. Rats in the OTA + RLE group showed portal spaces moderately expanded by fibrous connective tissue (blue) with MTRC staining. (**b**) Severity scores for inflammation, steatosis, necrosis, and fibrosis for each group. Asterisks represent statistically significant differences between groups (* *p* < 0.05, ** *p* < 0.01 vs control; ^#^ *p* < 0.05 vs OTA).

The livers collected from the four experimental groups underwent HE and MTRC evaluation (Figure 3a). Livers of rats in both the control and RLE groups exhibited mild to moderate lymphoplasmacytic inflammation as well as mild to severe steatosis. There was no evidence of necrosis or fibrosis in the livers of control and RLE rats, and no statistically significant changes were observed between control and RLE groups in terms of inflammation, necrosis, steatosis, and fibrosis. The frequency of central vein dilation and sinusoidal dilatation was found to be higher in the RLE group compared with the control group (Figure 3a).

The livers from the OTA group exhibited lymphoplasmacytic inflammation of mild to moderate intensity as well as moderate to severe steatosis and moderate to severe necrosis. Regarding fibrosis, moderate to severe fibrosis, frequently associated with the multifocal expansion of portal spaces, was observed in the livers of the OTA group. The severity of inflammation, necrosis, and fibrosis in the OTA group was shown to be significantly higher compared with both the control and RLE groups (*p* < 0.05) (Figure 3b). Central vein dilation and sinusoidal dilatation were observed with more occurrence in OTA-treated rats compared with the control.

Rats in the OTA + RLE group showed a mitigation of focal periportal inflammation compared with the OTA-intoxicated group. Despite the treatment with RLE, a mild steatosis persisted in OTA + RLE rats. However, a statistically significant reduction in liver necrosis and inflammation was observed in OTA + RLE rats when compared with those treated with OTA alone (*p* < 0.05). There were no significant differences in steatosis and fibrosis levels when comparing the OTA + RLE and OTA groups (Figure 3b). In addition, no significant variations in the occurrence of sinusoidal dilation were observed among the experimental groups.

## 4. Discussion

Contamination of food and feed with OTA has become an international issue in terms of food safety. Due to the highly challenging process required to remove OTA from the food chain, both animals and humans are routinely exposed. The scientific community, including professionals in the field of veterinary medicine, has recognized the importance of nutraceuticals and additives to animal feed for animal health [10], particularly since the prohibition of ionophore antibiotics by European Commission (Directive 1831/2003/CEE, 2003). As OTA OS is one of the main mechanisms of action of this toxin, nutraceuticals with antioxidant activity may serve as additives in preventing and mitigating the side effects of ROS imbalance during OTA exposure [10,20].

Recent in vivo investigations have provided evidence of the effectiveness of RLE in alleviating diabetic nephropathy [15] and OTA-induced nephrotoxicity [16]. Moreover, the protective effects of RLE have already been successfully tested as an oral additive for lambs, showing improved juiciness and reduced colour deterioration of their meat due to delayed lipid oxidation. As a result, this contributes to the production of healthier meat suitable for human consumption [21].

The liver has a crucial role in determining the productivity of animals, particularly in terms of meat and dairy production. Indeed, its good functionality contributes to favorable weight gains, enhanced fertility, and improved milk production [22]. Consequently, liver damage in animal production systems leads to significant economic implications. Moreover, as it is responsible for biotransformation processes, the liver is exposed to elevated concentrations of any mycotoxin in the bloodstream following oral ingestion, potentially leading to its impairment. Therefore, although the liver does not serve as primary target organ for OTA, it is still one of the most vulnerable organs to the harmful effects associated with its exposure. In fact, OTA metabolism and accumulation occur within the hepatocytes following intestinal absorption [23]. Since the effects of RLE against OTA toxicity in the liver have not yet been investigated, the present research has been designed to assess the efficacy of this extract to mitigate OTA-induced hepatotoxicity in vivo.

The determination of AST, ALT, and ALP enzymes in the blood serum has demonstrated its utility as a valuable diagnostic tool for assessing tissue injury. These enzymes have been particularly beneficial in liver toxicology investigations and have shown clinical significance under pathological conditions. In the present work, rats belonging to the OTA group were characterized by a marked increase in serum ALT, AST, and ALP, indicating the occurrence of hepatocyte damage. This finding aligns with the observations reported by Hassan et al. [24] and was further supported by histological analysis. Consequently, the OTA group underwent a decrease in overall protein concentrations. A similar pattern was observed in some fish species following OTA intoxication [25]. The observed phenomenon may be attributed to disturbance of protein biosynthesis caused by the toxicant. In addition, HE and MTRC staining performed on the OTA group revealed significant inflammation compared to the control, probably caused by OTA-induced leaky gut that, consequently, increased the entrance of LPS into the liver, facilitating the progression of liver inflammation [26].

RLE co-administration with OTA resulted in the recovery of liver injury in OTA-intoxicated rats. In fact, a significant decrease in the levels of transaminases was observed, along with a reduction in the amount of total protein. These findings indicate that, similarly to other natural hepatoprotective natural compounds [27], the natural extract under investigation exerts protective effects on the functional and antioxidant liver enzymes, which is also supported by the performed histological analysis.

In this study, MDA levels increased after OTA administration in agreement with the literature [14,28], suggesting lipid peroxidation with subsequent enhanced membrane permeability and impairment of membrane-bound protein functionality [29]. The increased lipid peroxidation correlated with the significant rise in hepatocytes’ cytonecrosis enzymes in sera, but also with reduction in SOD, CAT, and GPx activities in the livers of OTA rats. In fact, lipid peroxidation corresponds with a redox imbalance in the antioxidant systems and excessive free radical generation. The altered oxidative balance and the excessive production of free radicals has the potential to enhance the activation of additional transcription factors, resulting in the release of cytokines [30], as supported by the inflammation found in the histological analysis. In this context, the RLE ameliorated OTA-induced oxidative liver injury by reducing lipid peroxidation and improving the main activities of antioxidant enzymes, with a concomitant reduction in inflammation level. In fact, RLE seems to possess a hepatoprotective effect through the increase of key antioxidant activities, such as SOD, CAT, and GPx, while concurrently reducing inflammation. Although the inflammation has not been characterized, the evident reduction of the lymphocytic infiltrate in OTA-intoxicated rats treated with RLE shows the effectiveness of this extract, which deserves to be further investigated. However, the mitigation of OS by RLE could relate to the reduction of inflammatory status, as reported in Hussain et al. [31].

Susceptibility to OTA varies significantly depending on the species and the physiological stage. Monogastric animals have higher susceptibility to the impacts of OTA compared with polygastric species. This tendency arises from a combination of factors, including increased exposure to contaminated feed (mostly composed of cereals) and the absence of detoxification processes facilitated by the rumen [32]. Therefore, there is a pressing need to enhance the knowledge and understanding of livestock breeders regarding the issue of OTA contamination in animal feed. Additionally, it is imperative to emphasize the importance of implementing self-regulatory measures that effectively mitigate the possible risks associated with this mycotoxin. In this scenario, RLE could be considered a valid source for mitigating its effects, although its distribution kinetics need to be studied in the various livestock species. Furthermore, it is recommended to assess the influence of RLE on animals’ sexual behavior, considering that the present investigation focused exclusively on male rats. Additionally, it is important to consider the financial implications associated with including RLE in the diet of larger animals, as substantial quantities would be required.

Overall, the results presented in this work suggest that the protective role of RLE may be attributed to its ability to modulate the processes involved in the antioxidant defence mechanisms. While the exact mechanism of action of RLE remains unknown and needs further investigation, it is plausible to hypothesize that it might hinder the absorption of OTA. Hence, the use of RLE as a dietary supplement for animal feed could be a valid option. However, further evaluations are required to determine its suitability for use in feed, as well as its impact on the organoleptic properties of animals’ secondary products and its action against combinations of mycotoxins [33,34], which represent a more realistic scenario associated with the eating habits of both humans and animals.

## 5. Conclusions

This study reveals that RLE has a protective impact against OTA toxicity in the liver. This effect was demonstrated by a reduction in the levels of transaminases and total protein concentration, as well as an improvement in the redox imbalance that occurred after OTA exposure. As a result, RLE has the potential to be regarded as a biosustainable detoxifying molecule to be added to food and feeds for the purpose of preserving animal and human health from OTA oxidative imbalance.

## Figures and Tables

**Table 1:** Serum biochemical parameters: alanine aminotransferase (ALT), aspartate aminotransferase (AST), and alkaline phosphatase (ALP) activities expressed in units per liter (U/L) and total proteins expressed in grams per deciliter (g/dL). Control group (control); red orange and lemon extract group (RLE); ochratoxin A group (OTA); OTA plus RLE group (OTA + RLE). Data are expressed as mean ± standard deviation (SD) of *n* = 6 rats (**** *p* < 0.0001 vs. control; ^####^ *p* < 0.0001 vs. OTA).

Group	ALT (U/L)	AST (U/L)	ALP (U/L)	Total Protein (g/dL)
Control	43.29 ± 5.2	106.72 ± 1.41	24.45 ± 6.0	79.51 ± 3.40
RLE	38.07 ± 4.1	99.40 ± 5.8	20.48 ± 5.0	83.46 ± 1.42
OTA	105.24 ± 61 ****	185.88 ± 4.3 ****	91.28 ± 46 ****	48.15 ± 2.47 ****
OTA + RLE	81.23 ± 59 ^####^	54.69 ± 5.9 ^####^	61.26 ± 4.9 ^####^	82.09 ± 2.33 ^####^

## Data Availability

All of the data is contained within the article.
